# Elbow calcific tendinosis initially detected by ultrasonography: a case report

**DOI:** 10.1186/s13256-024-04381-x

**Published:** 2024-02-26

**Authors:** Masih Rikhtehgar, Yasaman Sharifi, Farid Najd Mazaher, Neda Azarpey

**Affiliations:** 1 Rasool_Akram Hospital, Radiology Department, Iran University of Medical Sciences, SattarKhan St., Tehran, Iran; 2 Endocrinology and Metabolism Research Center, Endocrinology and Metabolism Clinical Sciences Institute, Tehran University of Medical Sciences, Tehran, Iran; 3 Shafa Yahyaeian Hospital, Orthopedic Surgery Department, Iran University of Medical Sciences, Tehran, Iran

**Keywords:** Calcific tendinosis, Radiography, Ultrasonography, Subcutaneous lipoma

## Abstract

**Introduction:**

Calcific tendinosis is a common condition caused by the deposition of hydroxyapatite crystals within the tendons that can impact any tendinous insertion. In this situation, ultrasound (US) may be a viable imaging modality in addition to radiography.

**Case presentation:**

A 56-year-old Iranian male presented with left elbow pain. US evaluation at the lump site revealed a subcutaneous lipoma. Ultrasonography showed a thickened and diffusely heterogeneously hypoechoic pronator teres tendon. These characteristics were consistent with the diagnosis of calcific tendinosis.

**Discussion and conclusion:**

Radiography is the most common and practical imaging modality for calcific tendinosis diagnosis. Despite this, the real-time nature of ultrasonography makes it both diagnostic and therapeutic in this condition. Other conditions, such as lipoma, may interfere with the proper diagnosis of calcific tendinosis.

## Introduction

Calcific tendinosis is a common condition caused by the pathologic deposition of hydroxyapatite crystals within the tendons [1]. It mostly affects the shoulder, with a 2.7% adult population incidence rate [2, 3]. Although calcific tendinosis can appear in any tendinous insertion in the body [1], such as the hip, knee, wrist, finger, or elbow joints [3, 4]. This condition usually occurs in adults aged 30 to 60 and is also more common in women [1, 5]. It is imperative to note that calcific tendinosis of the elbow is a rare condition [3], but it also requires immediate attention to avoid a delayed or incorrect diagnosis. Radiography is the most practical method of evaluating calcific tendinosis [6]. Cost-effective and useful for detecting calcium deposits and for delineating and determining the extent and density of calcific tendinosis [7]. Alternative imaging modalities, such as sonography, computed tomography (CT) scans, or magnetic resonance imaging (MRI), may be used to confirm the diagnosis and rule out other possibilities [8–10]. A proper and immediate diagnosis could lead to better management of this condition for physicians or patients. A proper and prompt diagnosis may better manage this condition for physicians or patients. Hence, in this rare case, we have a patient with pain located on the medial part of the left elbow and a sensation of a lump in this location, both of which were investigated with imaging modalities to confirm the diagnosis of calcific tendinitis.

## Case presentation

A 56-year-old Iranian male (right-handed) presented with left elbow pain that lasted for three months. In addition to feeling pain, the patient also complained of a palpable lump.

**Fig. 1:**
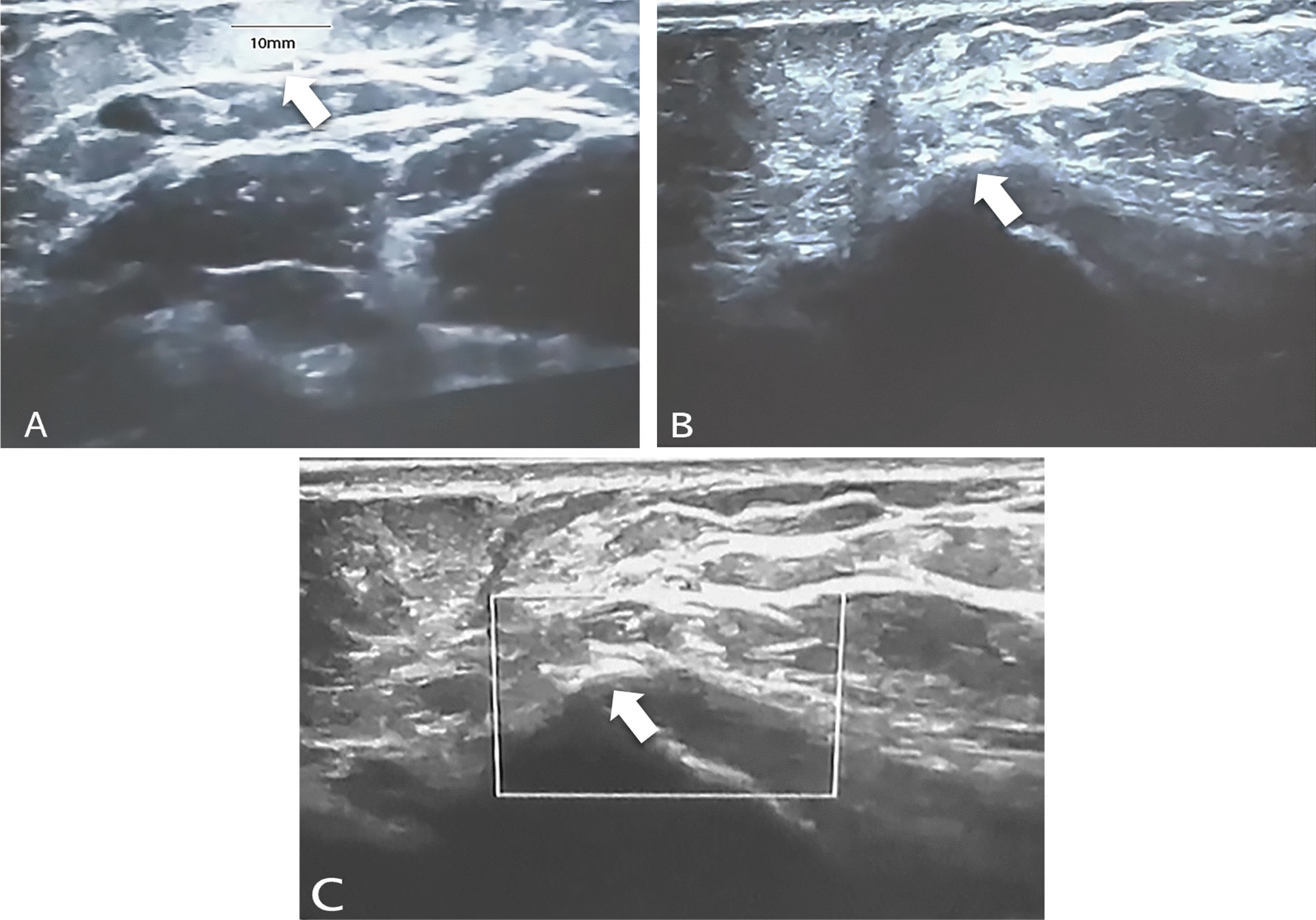
The ultra-sonographic imaging of patients with pain and a non-tender mass in the left elbow. **A** a well-defined, compressible hyperechoic lesion with a maximum diameter of 10 mm that is consistent with a subcutaneous lipoma (Arrow). **B** A US scan of the pronator teres tendon origin site revealed a thicker and more diffusely heterogeneous tendon with a dense hyperechoic calcification site, suggesting calcific tendinitis(Arrow). **c** On color Doppler ultrasound, there is no flow at the calcified site at the origin of the pronator teres tendon (Arrow)

**Fig. 2:**
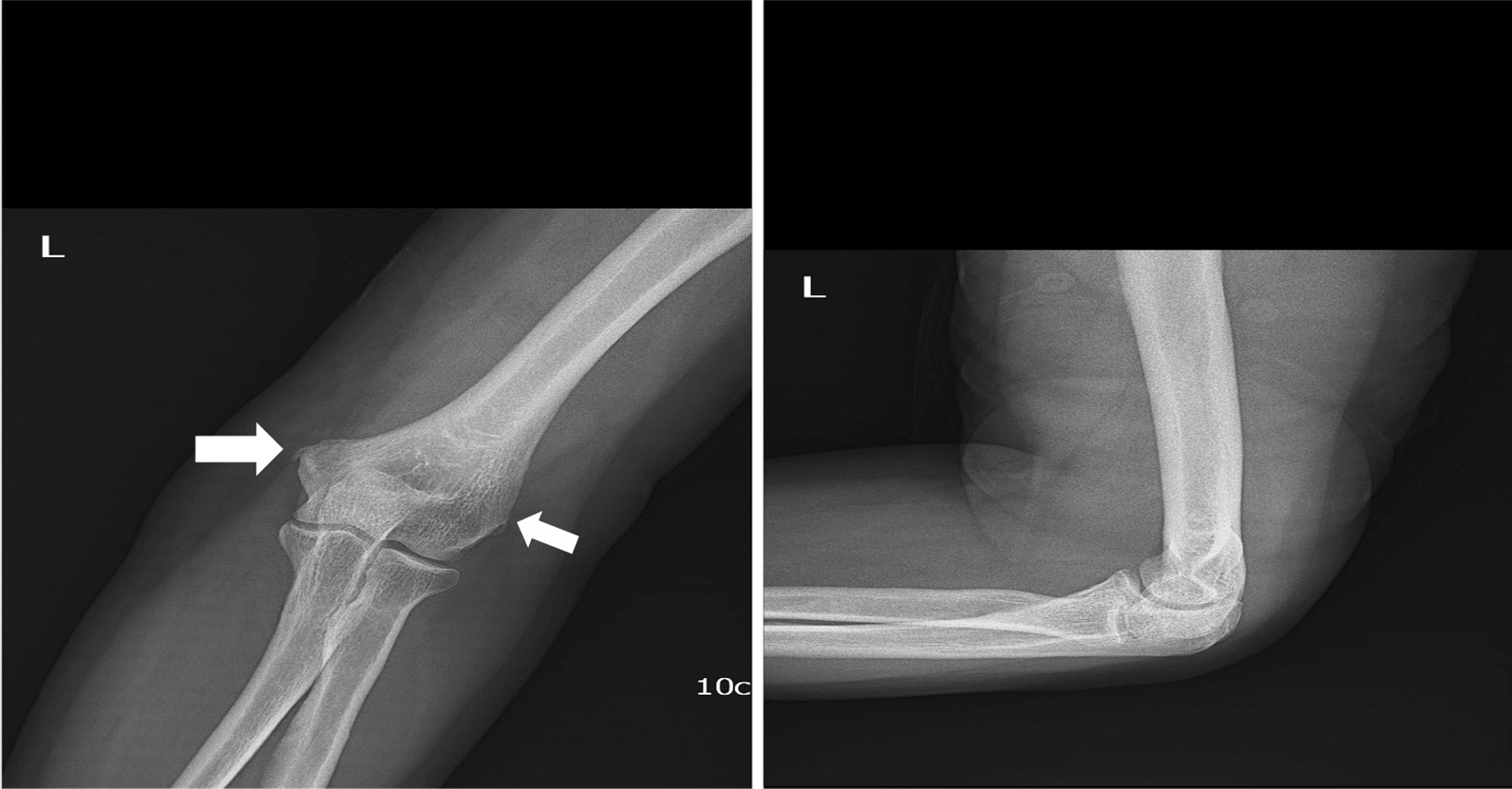
Anterior–posterior and lateral view radiographs of the left elbow. The imaging shows the presence of a small calcification in the soft tissues near both epicondyles mainly in the lateral epicondyle (arrow)

Following the physical examination, he revealed that he has been unable to fully extend his elbow since last month, and that he has tenderness on the medial epicondyle of his left elbow. There was no previous history of elbow injuries. His family history was unremarkable. The patient also revealed no medical or surgical history. The orthopedic surgeon examined him and found that he had a tender site as well as a non-tender lump over the medial epicondyle region of the humerus. An MRI was taken, which showed a lipoma and non-specific inflammatory change at the site of marker placement. The patient was referred to us by a radiologist for an ultrasound to further investigate the MRI findings as a probable explanation of the patient's chief complaints. Sonographic imaging at the site of pain and complaints with a linear probe was done. Matrix linear-array transducers with a peak frequency of 4 to 15 MHz were used for this case (with the GE Voluson E6 Ultrasound Machine). The origin of the concern was examined with ultrasound. There was a 10 mm round-shaped well-defined hyperechoic subcutaneous lesion that was congruent with the location of the lump sensation but with no pain, consistent with subcutaneous lipoma, adjacent to the left medial epicondyle of the elbow (Fig. 1A). Further examination with ultrasound at the site of pain revealed a thickened and diffusely heterogeneously hypoechoic pronator teres tendon, with calcific deposition (arrow) and notably uneven underlying bone (Fig. 1B). On color or power Doppler imaging revealed no evidence of hypervascularity in the region of calcification at the origin of the pronator teres tendon (Fig. 1C). These characteristics were consistent with the diagnosis of calcific tendinosis and subcutaneous lipoma at the same time and site. An X-ray scan of the patient's left elbow was obtained based on this suspected diagnosis. The plain radiograph revealed heterotopic bone density in both the medial and lateral humeral epicondyles, but predominantly in the lateral epicondyle, which conflicted with the patient's clinical complaints (Fig. 2). These imaging modalities validated the diagnosis of calcific tendinosis, and no additional assessment was required. The patient was referred to his orthopedic surgeon for treatment based on the results of the US examination and X-ray findings. The orthopedic physician recommended conservative treatment for him as well as a two-month follow-up visit with a physical examination.

Considering the heterotopic bone density on both sides of the epicondyles, which could be indicative of calcific tendinosis on both sides, an X-ray of additional probable sites, as well as blood testing to rule out any endocrine abnormalities, was ordered.

Upon the follow-up appointment, the patient’s laboratory testing indicated no abnormalities, such as hyperparathyroidism or other endocrine disorders that may cause hypercalcemia. Other X-rays (Knee/Shoulder/Other elbow) showed no other abnormalities and mainly no calcific tendinosis.

## Discussion

Herein, a case of calcific tendinosis of the medial condyle of the humerus is presented. As a first step, the orthopedic surgeon examined the patient and found that he had a palpable mass simultaneously in approximately the same location, and the radiologist performed an ultrasound instead of normal X-rays. An ultrasound assessment of this affected person found calcific tendinosis and subcutaneous lipoma.

Calcific tendinosis is considered to be prevalent in the shoulder, and it usually affects a single joint. In the general population, its prevalence varies significantly according to its location in the body (3–22%) [11]. It is a relative rarity to develop calcific tendinosis of the elbow (medial or lateral condyle) [4]. Previous studies have reported that calcific tendinosis is more prevalent among females [2, 12]. The mean incidence age is 47 and 51 years among females and males, respectively, according to the Lippmann study [12]. In our case, the unique presentation of a man with medial humeral epicondyle pain and a lump sensation could have directed the patient to a radiologist for an ultrasound instead of a plain x-ray, and the diagnosis of calcific tendinosis of the elbow could be a rare differential diagnosis.

Although calcific tendinosis is often asymptomatic in many patients, it can cause significant joint pain [1, 2]. The patient's primary complaint was pain in the medial part of his elbow. Additionally, he felt a lump at this location, which resulted in him being unable to fully extend his arm.

Calcific tendinosis is a dynamic condition that advances in stages. Stages have been identified as having different radiographic and pathological characteristics, which frequently coincide with clinical symptoms [1]. The deposition of fluffy and amorphous calcifications represents the resorption phase of calcific tendinitis, which is commonly accompanied by pain and reduced mobility [1]. In this stage, patients may be suspected of having other pathologies, such as septic arthritis and fractures [1]. As in our case, this stage could explain his clinical complaints and radiographic evaluation since he complained of pain and decreased range of motion in his left elbow compared to the other site. In this stage, imaging findings of calcific tendinosis may involve severe osseous changes and substantial soft-tissue edema. These results are most commonly seen in cross-sectional modalities such as CT and MRI [1, 9]. Characterizing the shape and contour of the calcific deposit is crucial [1]. Although deposits with fluffy, hazy, ill-defined borders are frequently detected in individuals with acute pain and may be associated with the resorptive phase of calcific tendinitis, deposits with a well-defined, homogenous contour are less likely to be symptomatic and may be associated with the formative or calcific phases [13].

The use of ultrasound can help diagnose calcific tendinitis, especially in the shoulder [8]. The real-time nature of this imaging modality makes it both diagnostic and therapeutic [1, 8, 14]. Tendon calcification manifests as a hyperechoic center with or without posterior acoustic shadowing [1, 15]. Ultrasound can accurately detect the exact location of calcification, especially in the rotator cuff tendon, although it has limits in assessing the pathophysiologic phase. As a result, sonography should be done in combination with radiographs to appropriately determine the probability of other pathologic bone conditions [1, 15, 16].

To the best of our knowledge, there haven't been any documented cases of calcific tendinosis in the elbow assessed with sonographic imaging as a first modality. In a study by Ferin et al.[15], sonographic imaging has been used to diagnose calcific tendinosis of the shoulder and rotator cuff tendons. The sonographic findings of this study include several forms of calcification, such as a hyperechoic focus with a well-defined shadow (79%), a hyperechoic focus with a faint shadow (14%), and a hyperechoic focus with no shadow (7%) [15]. Another study looked at the ultrasonography findings of lateral epicondylitis of the elbow [17]. According to this study, the ultrasound findings of the common extensor tendon had high sensitivity but limited specificity in detecting symptomatic lateral epicondylitis. In addition, there was a statistically significant correlation between symptoms and intra-tendinous calcification, tendon thickening, surrounding bone irregularity, focal hypoechoic areas, and diffuse heterogeneity [17].

In our case, the coincidence of a subcutaneous lipoma at the same location as the patient's pain may mislead the diagnosis of calcific tendinosis if the patient performed an MRI or CT scan of his affected elbow since the location was compatible with the pain site and the reduced range of motion may also be explained by the lipoma, although the ultrasound evaluation as a real-time imaging modality reported the subcutaneous lipoma as an accidental finding and the calcification of the pronator teres origin of the tendon as a primary diagnosis and the leading cause of the patient's pain and reduced mobility of the elbow. These findings were confirmed by the radiographs, which show the importance of musculoskeletal ultrasonography in the combination of plain radiographic imaging in the diagnosis of calcific tendinosis, like any other soft tissue calcification disorder.

The first line of treatment for painful calcific tendinosis is usually conservative, consisting of nonsteroidal anti-inflammatory medications, rest, and physical therapy [15]. Although the process is self-limiting, many individuals may be unable to tolerate the length of time required for resolution; hence, invasive treatment is performed in these circumstances [8]. Since there was no indication for invasive treatment in our case and the diagnosis was neither delayed nor misdiagnosed, conservative treatment was also considered for him.

## Conclusions

Calcific tendinosis is a rare ailment that affects the elbow joint; as a result, diagnosis may be delayed at this site. This entity necessitates a great degree of suspicion and alertness. In this case, we could observe that sonographic imaging and plain radiographic assessment are both reliable and effective. Since other modalities, such as MRIs and CTs, are so easily accessible, this modality is infrequently used. Nonetheless, when paired with plain radiographic imaging, the efficiency of this modality is preserved, especially when alternative diagnoses such as soft tissue masses are suspected.

## Learning points

1. Calcific tendinosis is a common disease that can affect any tendon origin in the body, but it most commonly affects the shoulder tendons.
2. The first modality for the diagnosis of calcific tendinosis is plain radiographs.
3. Real-time ultrasound imaging, particularly in cases where multiple possible causes of pain or movement limitation exist, could aid in determining the exact source of the problem and reaching a diagnosis and could be a potential imaging modality in calcification deposits in musculoskeletal diseases.

## Data Availability

Data sharing does not apply to this article as no datasets were generated or analyzed during the current study.
